# Case Report: Two New Cases of Autosomal-Recessive Hypertrophic Cardiomyopathy Associated With *TRIM63*-Compound Heterozygous Variant

**DOI:** 10.3389/fgene.2022.743472

**Published:** 2022-02-22

**Authors:** Sofiya Andreeva, Olga Chumakova, Elena Karelkina, Viktoriya Lebedeva, Tamara Lubimtseva, Andrey Semenov, Alexey Nikitin, Gleb Speshilov, Alexandra Kozyreva, Polina Sokolnikova, Sergey Zhuk, Yuliya Fomicheva, Olga Moiseeva, Anna Kostareva

**Affiliations:** ^1^ Institute of Molecular Biology and Genetics and World-Class Research Centre for Personalized Medicine, Almazov National Medical Research Centre, Saint Petersburg, Russia; ^2^ Central State Medical Academy of Department of Presidential Affairs, City Clinical Hospital #17, Moscow, Russia; ^3^ Institute of Heart and Vessels, Almazov National Medical Research Centre, Saint Petersburg, Russia; ^4^ Pulmonology Research Institute, Federal Medical-Biological Agency of Russia, Moscow, Russia; ^5^ Laboratory of Genotyping, N. F. Gamaleya National Research Center, Moscow, Russia; ^6^ Department of Women’s and Children’s Health and Center for Molecular Medicine, Karolinska Institute, Solna, Sweden

**Keywords:** hypertrophic cardiomyopathy, *TRIM63*, MuRF1, compound heterozygote, extreme hypertrophy, diastolic dysfunction

## Abstract

Hypertrophic cardiomyopathy (HCM) is one of the most common hereditary diseases, and it is associated with fatal complications. The clinical heterogeneity of HCM requires risk prediction models to identify patients at a high risk of adverse events. Most HCM cases are caused by mutations in genes encoding sarcomere proteins. However, HCM is associated with rare genetic variants with limited data about its clinical course and prognosis, and existing risk prediction models are not validated for such patients’ cohorts. *TRIM63* is one of the rare genes recently described as a cause of HCM with autosomal-recessive inheritance. Herein, we present two cases of HCM associated with *TRIM63*-compound heterozygous variants in young male sportsmen. They demonstrated progressively marked hypertrophy, advanced diastolic dysfunction, a significant degree of fibrosis detected by magnetic resonance imaging, and clear indications for implantable cardioverter-defibrillator. One of the cases includes the first description of *TRIM63*-HCM with extreme hypertrophy. The presented cases are discussed in light of molecular consequences that might underlie cardiac and muscle phenotype in patients with mutations of *TRIM63*, the master regulator of striated muscle mass.

## Introduction

Hypertrophic cardiomyopathy (HCM) is the most common cardiovascular hereditary disease, and it is characterized by cardiac hypertrophy that cannot be explained solely by abnormal loading conditions (Ommen et al., 2020). The history of genetic research in HCM goes back to 3 decades, and up to date, over 450 causative mutations in at least 20 genes encoding for sarcomeric and myofilament-related proteins have been described (Geisterfer-Lowrance et al., 1990; Wolf, 2019). Mutations in *MYBPC3* and *MYH7* are the most frequent genetic cause of HCM and attributed to more than 50% of all HCM cases. Other HCM causal mutations have been identified in TNNT2, TNNI3, and genes encoding for structural proteins (Sabater-Molina et al., 2018; Teekakirikul et al., 2019). However, in 30–40% of the cases, the origin of the disease still remains unclear despite extensive genetic testing using targeted gene panels (Wolf, 2019; Salazar-Mendiguchía et al., 2020). In the case of HCM associated with rare causative genes, the existing risk prediction models may not be accurate enough because they have not been validated for this particular population. Therefore, collecting clinical and genetic data of patient groups with the rare genetic background of HCM may facilitate the development of more accurate and personalized risk stratification algorithms.

*TRIM63* (tripartite motif 63) is a gene only recently described in association with HCM (Chen et al., 2012; Salazar-Mendiguchía et al., 2020). *TRIM63* encodes muscle-specific RING-finger protein 1 (MuRF1), a member of ubiquitin ligases subfamily, such as MuRF-2 and MuRF-3.

In skeletal myocytes, upregulation of MuRF1 underlies a broad spectrum of muscle atrophy conditions (Peris-Moreno et al., 2020). In cardiac myocytes, overexpression of MuRF1 enhances susceptibility to heart failure in response to pressure overload, and its activation prevents cardiac hypertrophy (Arya et al., 2004; Willis et al., 2009). MuRF1 targets include sarcomere contractile, structural proteins, and signaling molecules (Witt et al., 2005; Polge et al., 2011; Chen et al., 2012; Maejima et al., 2014; Polge et al., 2018; Higashikuse et al., 2019; Bulatov et al., 2018). The involvement of *TRIM63* in various pathological processes has been documented in several experimental and functional studies (Chen et al., 2012; Su et al., 2014; Olivé et al., 2015; Salazar-Mendiguchía et al., 2020).

The original study of Chen et al. revealed three *TRIM63* variants in five unrelated probands among 302 probands with HCM*.* Additionally, they identified two loss of function variants (p.Ala48Val and p.Ile130Met) and one protein-truncation variant (p.Gln247*) detected in the heterozygous state. Experimental cellular and animal studies demonstrated the reduced colocalization of MuRF1 with alfa-actinin at the Z-disk level, impaired auto-ubiquitination, and depressed ubiquitination and proteasome degradation of substrates (Chen et al., 2012).

Later, the role of p.Gln247* as a single causative variant leading to HCM was challenged due to its relatively frequent detection in healthy European subjects and observation of its clinical phenotype only in the cases of compound heterozygosity or homozygous variants (Płoski et al., 2014). Su et al. found rare variants in MuRF1 and MuRF2 encoding genes in the healthy population but with a much lower frequency than in HCM. Moreover, carriers of these variants had greater maximum left ventricular wall thickness than non-carriers. Hence, rare variants in MuRF1 and MuRF2 encoding genes were associated with higher penetrance and more severe clinical manifestations of HCM, especially when coincided with other sarcomeric mutations, therefore being considered by the authors as modifiers in HCM. It is important to note that all detected rare variants were heterozygous (Su et al., 2014).

The final clarification of the *TRIM63* inheritance pattern became possible after studying 4,867 index cases with HCM (Salazar-Mendiguchía et al., 2020). *TRIM63* sequencing and subsequent familial evaluation revealed that only homozygous and compound heterozygous carriers developed full clinical phenotype of the disease (15 patients with HCM and one with restrictive cardiomyopathy), while heterozygous individuals demonstrated mild or almost no phenotype. These data strongly support the hypothesis that *TRIM63* causes HCM with autosomal-recessive inheritance (Salazar-Mendiguchía et al., 2020).

Herein, we present two cases of *TRIM63-*associated HCM due to compound heterozygous mutations along with results of a 5-year follow-up.

## Case Description

### Patient 1

**FIGURE 1:**
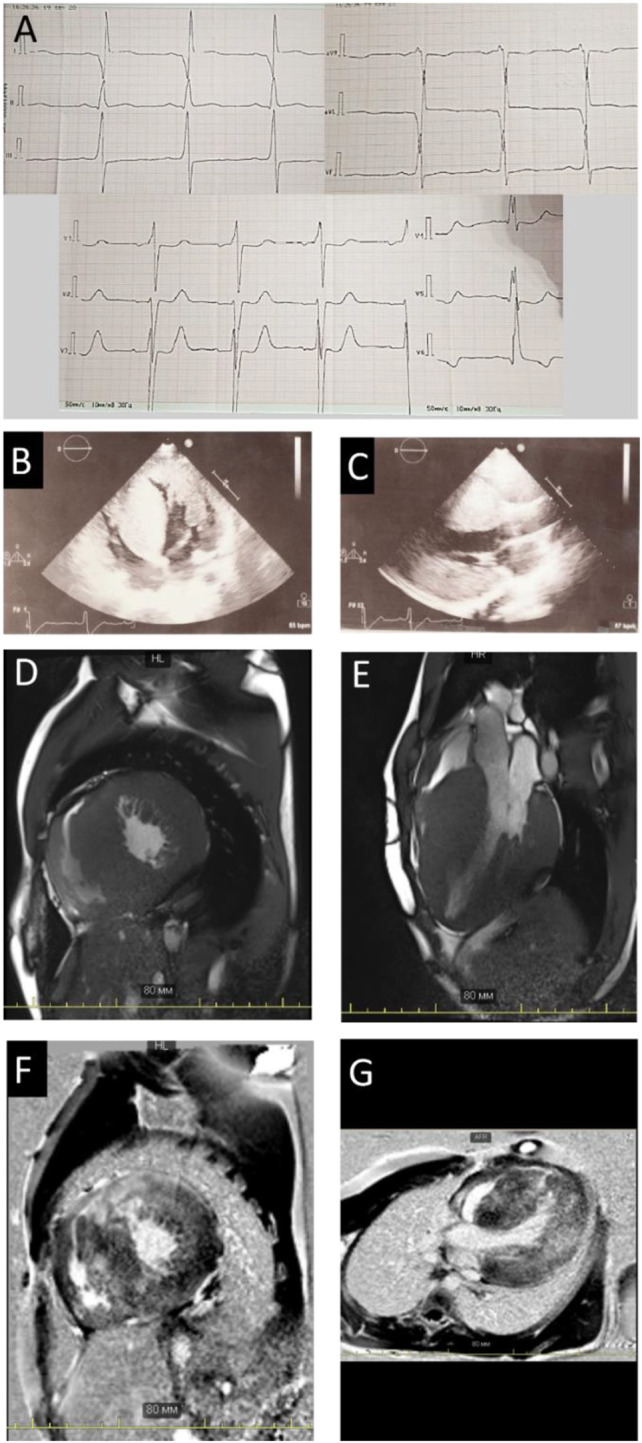
Instrumental findings in Patient 1. Electrocardiogram **(A)** and echocardiography picture correspondence to four-chamber **(B)** view and long-axis view **(C)** illustrated severe hypertrophy. Сardiac MRI images in the short **(D)** and long **(E)** axes, demonstrating wall hypertrophy, limited cavity volume, and late gadolinium enhancement **(F,G)**.

A nineteen-year-old male patient was hospitalized due to ECG and echocardiography abnormalities detected during scheduled examination for military enlistment. The patient denied any chest pain, dyspnea, palpitations, headache, dizziness, or syncope. His medical history was notable for hypertension (maximum blood pressure of 140/90 mmHg), without antihypertensive therapy. His exercise tolerance was high, and he had been performing weightlifting for several years. The patient had normal childhood development and demonstrated normal intelligence. He denied smoking and alcohol abuse and did not have a family history of sudden cardiac death (SCD) or early cardiovascular diseases. Physical examination revealed increased body mass index—25 kg/m^2^ and prominent hypertrophy of upper limb muscles. ECG and Holter monitoring demonstrated sinus rhythm, signs of biventricular hyperthrophy (Cornell voltage criteria is about 50 mm, R wave in ^1^V 10 mm), and prolonged QT interval (QTc up to 563 ms on Holter) (Figure 1A). Echocardiography showed extreme asymmetric left ventricular (LV) concentric hypertrophy with a predominant increase of interventricular septum (up to 48 mm) without left ventricle outflow tract obstruction (LVOT), right ventricle (RV) hypertrophy, mild left atrium dilatation, and restrictive type of diastolic dysfunction without signs of pulmonary hypertension (Table 1; Figures 1B,C).

**TABLE 1:** Dynamics of echocardiography data in Patient 1.

Parameter/age of examination	19 years old	23 years old	25 years old
LA, mm	46	50	48
LA volume, ml	68	60	126
LA volume index, ml/m^2^	30	26	43
RA, mm	44*46	40*45	44*52
Septum, mm	48	50	50
PW, mm	18	37	45
RWT	0.76	1.54	2.0
LV mass, g	1,129	2017	2,900
LV mass index, g/m^2^	495	878	988
LV EDD, mm	47	48	48
LV ESD, mm	31	29	27
LV EDV, ml	120	151	105
LV ESV, ml	42	63	28
SV, ml	78	88	77
EDVi, ml/m^2^	52.6	65.7	35.7
ESVi, ml/m^2^	18.4	27.4	9.5
RV WT, mm	9	11	11
RV, mm	28	29	42
EF, % (Simpson)	65	58	70
GL strain, %	—	-	-
TAPSE, cm	>1.6	2.2	>1.6
ePASP, mmHg	18–23	ND	35
Diastolic dysfunction, type	III	II	II
E/A ratio	2.02	1.7	1.82
LVOT PGmax, mmHg	10.68 at rest. Without increase after Valsalva maneuver	84 at rest	37 at rest during sinus rhythm, 97 during extrasystole
RVOT PGmax, mmHg	—	39	—
Mitral regurgitation	—	Mild	Mild
SAM of the MV	—	+	-

EDV, end-diastolic volume; EDVi, end-diastolic volume index; EF, left ventricle ejection fraction; ESVi, end-systolic volume index; ePASP, estimated pulmonary artery systolic pressure; GL strain, global longitudinal strain; LA, left atrium; LV EDD, left ventricle end-diastolic dimension; LV EDV, left ventricle end-diastolic volume; LV ESD, left ventricle end-systolic dimension; LV ESV, left ventricle end-systolic volume; LVOT PGmax, left ventricle outflow tract maximum pressure gradient; PW, posterior wall; RA, right atrium; RV, right ventricle; RVOT PGmax, right ventricle outflow tract maximum pressure gradient; RV WT, right ventricle wall thickness; RWT, relative wall thickness; SAM of the MV, the systolic anterior motion of the mitral valve; SV, stroke volume; TAPSE, tricuspid annular plane systolic dysfunction.

Stress-echocardiography excluded dynamic obstruction of LVOT (maximum pressure gradient 19 mmHg upon exertion). According to the European model “HCM Risk-SCD Calculator,” the 5-year risk of SCD was estimated as 3.6%. Therefore, an implantable cardioverter-defibrillator (ICD) implantation was not indicated, and the patient was discharged on metoprolol succinate (25 mg daily). Two years later, he noticed brief episodes of palpitations resulting in clinical re-evaluation at the age of 23. Echocardiography documented an increase in LV and RV wall thickness (up to 50 and 11 mm, resp.), mitral valve systolic anterior motion, and LVOT and RV outflow tract obstruction at rest (Table 1; Figure 1 B,C). According to magnetic resonance imaging (MRI), circularly intramurally late gadolinium enhancement (LGE) was documented (Figures 1D–G; Supplementary Table S1). Re-evaluation of the 5-year risk of SCD resulted in a 4.94% probability, a dual-chamber ICD was implanted, and metoprolol therapy was continued.

During the next 2 years, a progressive increase in myocardial mass, predominantly due to posterior wall thickness, was observed (Table 1). Holter monitoring demonstrated multiple single and paired polymorphic ventricular extrasystoles and episodes of non-sustained polymorphic ventricular tachycardia but no appropriate ICD discharges.

To uncover the molecular background of the disease, targeted genetic testing was performed using a SureSelect panel of 108 cardiomyopathy-associated genes for Patient 1 (Supplementary Table S2), and no disease-related variants were detected. A subsequent whole-exome sequencing for Patient 1 was performed as described previously using a SureSelect Human All Exon V6 r2 (60 Mbp) target enrichment kit (Agilent Technologies, Santa Clara, CA, United States) with an Illumina HiSeq instrument and SBSv4 chemistry (Vershinina et al., 2020). Data curation, alignment strategy, variant calling, and filtering were performed according to GATK BestPractice recommendation using hg19 reference and annotated with ANNOVAR as previously published (Jorholt et al., 2020). Raw sequencing data are deposited at the SRA database under the reference number SRR16609854. Data interpretation was performed according to the guidelines of the American College of Medical Genetics (ACMG) (Richards et al., 2015). This resulted in the detection of two variants in the TRIM63 gene (NM_032588: c.T115G:p.C39G and NM_032588:c.481_482del:p.S161CfsTer8) in compound heterozygous form (Figure 3A; Table 2). The first variant, C39G, is newly described, absent in all available databases, and currently, according to ACMG, classified as a variant of unknown significance. A second variant, S161CfsTer8, has been described in a patient with HCM, reported in the GnomAD database (rs540072010), while being classified as a variant of unknown significance according to ACMG has been reported in ClinVar as pathogenic. No pathogenic or likely pathogenic variants or variants of unknown significance were detected in the genes causing storage diseases. Unfortunately, the parental DNA was not available for the analysis due to the parental refusal to participate in genetic analysis and *de novo*/inherited status of the variants remained unknown (Figure 3B).

**TABLE 2:** Genetic variants in *TRIM63* gene of Patient 1 and Patient 2.

Patient	Pathogenicity		Gene	Position GRCh37 and nomenclature	rs	MAF,%
	ACMG	ClinVar				
1	VUS	—	*TRIM63*	Chr1: 26393871:A>C	—	—
				NM_032588: c.T115G:p.C39G		
	VUS	Pathogenic	*TRIM63*	Chr1: 26387675:ACT>A	rs540072010	0.004
				NM_032588:c.481_482del:p.S161CfsTer8		
2	VUS	Likely pathogenic	*TRIM63*	Chr1: 26384973G>A	rs14839503	0.07
				NM_032588: c.739C>T: p.Q247X		
	VUS	—	*TRIM63*	Chr1: 26392867C>T	rs200811483	0.01
				NM_032588: c.224G>A: p.C75Y		

ACMG, American College of Medical Genetics; MAF, minor allele frequency; VUS, variant of unknown significance.

### Patient 2

**FIGURE 2:**
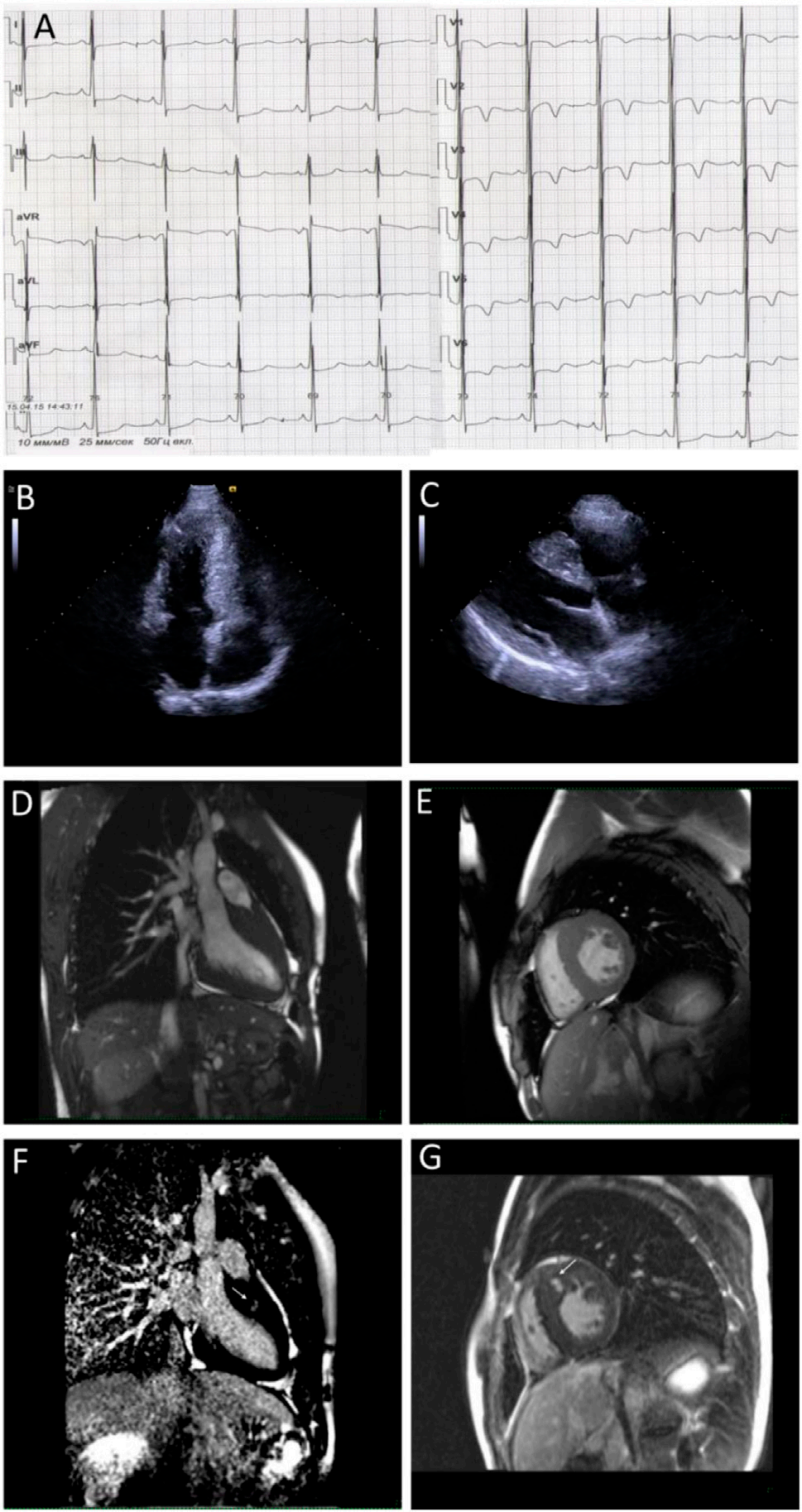
Instrumental findings of Patient 2. Electrocardiogram demonstrates voltage criteria of left ventricle hypertrophy and secondary repolarization changes **(A)**. Echocardiogram correspondence to four-chamber **(B)** and long-axis view **(C)** and MRI images in the long **(D)** and short-axis view **(E)** confirm wall hypertrophy. Arrows indicate the late gadolinium enhancement phenomenon **(F,G)** in the basal anteroseptal segment.

A fifteen-year-old male, a professional soccer player, was examined due to the first syncope that occurred during a sports activity. Conventional ECG revealed ST-segment elevation and T wave inversions up to 6 mm in all precordial, I, and aVL limb leads; signs of severe LV hypertrophy (Sokolow–Lyon index was 66 mm); and borderline QTc interval (480 msec) (Figure 2A). Echocardiography demonstrated asymmetric LV hypertrophy with a maximal wall thickness of 16 mm in basal and middle anteroseptal segments without LVOT obstruction at rest and after the exercise stress test (Figures 2B,C). The cardiac MRI using gadolinium enhancement demonstrated fibrosis, but Holter monitoring did not reveal ventricular arrhythmia or conduction abnormalities (Figures 2D–G). His body mass index and physical and intellectual development were normal, and no signs of peripheral myopathy were noted. The family history was free from SCD episodes, and parental ECG and echocardiography were normal. Over the next 9 years, the patient has been involved in active sport despite restrictive recommendations and remained asymptomatic. Echocardiography detected the progressive increase in septal and anterior wall thickness without LVOT obstruction and decreased LV cavity, and the appearance of LV diastolic dysfunction with mild enlargement of the left atrium was observed (Table 3). According to the ESC calculator, the estimated 5-year risk of SCD was high (7.8%) despite unremarkable Holter monitoring, but the patient refused the ICD implantation and remained only on metoprolol therapy.

**TABLE 3:** Dynamics of echocardiography data in Patient 2

Parameter/age of examination	15 years old	19 years old	22 years old
LA, mm	39	45	47
LV volume, ml	60	61	68
LA volume index, ml/m^2^	33	32	36
RA volume, ml	49	51	63
RA volume index, ml/m^2^	27	27	34
Septum, mm	16	24	27
PW, mm	11	11	11
RWT	0.48	0.46	0.44
LV mass, g	335	—	—
LV mass index, g/m^2^	184	—	—
LV EDD, mm	46	48	50
LV ESD, mm	30	—	23
LV EDV, ml	114 (Teicholz)	71	79
LV ESV, ml	50 (Teicholz)	19	24
SV, ml	64	52	55
EDVi, ml/m^2^	62.6	37.8	42.0
ESVi, ml/m^2^	27.5	10.1	12.8
RV WT, mm	4	4	4
RV, mm	25	26	31
EF, %	57	69	70
GL strain, %	19.3	20	—
TAPSE, cm	—	—	—
ePASP, mmHg	11	—	24
Diastolic dysfunction, type	No	No	II
E/A ratio	1.53	1.4	1.6
LVOT PGmax, mmHg	4	7	7
RVOT PGmax, mmHg	—	—	—
Mitral regurgitation	mild	mild	mild
SAM of the MV	No	No	No

EDV, end-diastolic volume; EDVi, end-diastolic volume index; EF, left ventricle ejection fraction; ESVi, end-systolic volume index; ePASP, estimated pulmonary artery systolic pressure; GL strain, global longitudinal strain; LA, left atrium; LV EDD, left ventricle end-diastolic dimension; LV EDV, left ventricle end-diastolic volume; LV ESD, left ventricle end-systolic dimension; LV ESV, left ventricle end-systolic volume; LVOT PGmax, left ventricle outflow tract maximum pressure gradient; PW, posterior wall; RA, right atrium; RV, right ventricle; RVOT PGmax, right ventricle outflow tract maximum pressure gradient; RV WT, right ventricle wall thickness; RWT, relative wall thickness; SAM of the MV, systolic anterior motion of the mitral valve; SV, stroke volume; TAPSE, tricuspid annular plane systolic dysfunction.

**FIGURE 3:**
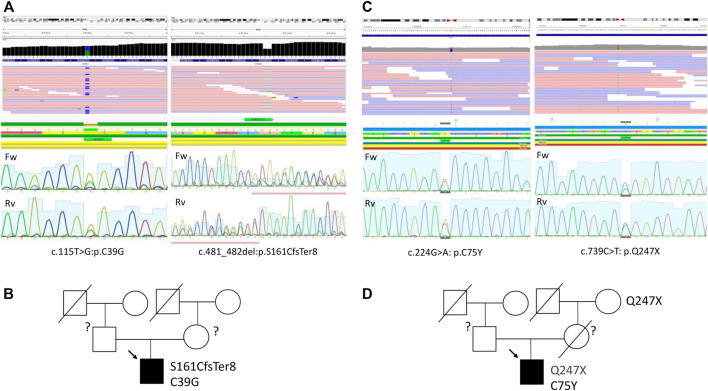
Sequencing data and pedigree of Patient 1 **(A,B)** and Patient 2 **(C,D)**.

A targeted sequencing using 176 cardiomyopathy-associate gene panel (Supplementary Table S3) resulted in the detection of two variants in the TRIM63 gene (NM_032588: c.739C>T: p.Q247X and NM_032588: c.224G>A: p.C75Y) in compound heterozygous form (Table 2; Figure 3C), and raw sequencing data are deposited at SRA database under the reference number SRR16946091. Both variants are present in GnomAD, have been previously reported in association with HCM in compound heterozygous form, and, according to ACMG, are classified as a variant of unknown significance. Similar to the previous case, no potentially causative variants were detected in the genes causing storage diseases. The parental DNA was not available for the analysis as the contact to the patient’s parents was lost and he was brought up solely by his maternal grandmother. However, the Q247X variant was confirmed to be present in the maternal grandmother with no clinical and echocardiography signs of HCM (Figure 3D).

## Discussion and Conclusion

The presented cases describe the clinical phenotype of *TRIM63*-associated HCM and extend our knowledge on rare genetic forms of one of the most common inherited human disorders. While HCM remains the most common genetically predicted cardiovascular disease, the vast majority of the cases are linked to the variants in eight sarcomeric genes (*MYBPC3*, *MYH7*, *ACTC*, *TTNI3*, *TTNT2*, *TPM1*, *MYL2*, and *MYL3*) with a clear predominance of *MYH7* and *MYBPC3* variants. Therefore, most clinical algorithms and guidelines are developed using these cohorts of patients (Ho et al., 2018; Lorenzini et al., 2020). Rare genetic forms of HCM account for only 1–2% of the cases (Lopes and Elliott, 2014). However, despite the small number of patients with rare variants, their total number makes up almost one-third of all patients with sarcomeric mutations (Ingles et al., 2019). Currently, it is not clear to what extent the common guidelines and recommendations are valid for these rare genetic groups of HCM. It is generally accepted that HCM associated with sarcomere gene mutations has a worse prognosis than non-sarcomeric forms of the disease (Ho et al., 2018; Marstrand et al., 2020). However, several exceptions exist, such as HCM, associated with *PRKAG2* gene mutations (Ahamed et al., 2020). Thus, such rare clinical variants are waiting for more cases reported along with broader clinical and prospective descriptions.

One of the rare HCM genetic variants is a form of the disease associated with the *TRIM63* gene. It has been described recently and represents one of the rare forms of autosomal-recessive or compound heterozygous form of HCM (Salazar-Mendiguchía et al., 2020). Despite the small number of cases reported by now, several characteristic features of the disease could distinguish this form from the “classical” sarcomeric phenotype. These specific characteristics include the relatively rapid increase in LV free wall thickness, the appearance of diastolic dysfunction from II to III grades with mild atria enlargements, and normal or borderline estimated pulmonary artery systolic pressure. For example, Patient 1 had extreme hypertrophy (septum up to 56 mm), progressing mainly due to posterior wall and RV wall thicknesses, shaping LVOT and RV outflow tract dynamic obstruction. To the best of our knowledge, it is the first report of *TRIM63*-associated HCM with such high magnitude hypertrophy (Chen et al., 2012; Su et al., 2014; Olivé et al., 2015; Salazar-Mendiguchía et al., 2020).

The diastolic dysfunction up to restrictive phenotype was already reported in *TRIM63*-mutation carriers by other authors (Olivé et al., 2015; Salazar-Mendiguchía et al., 2020). While not rare in some forms of HCM, restrictive phenotype had a malign prognosis in pediatric and adult patients with HCM (Maskatia et al., 2012; Li et al., 2020). In *TRIM63*-associated HCM, restrictive dysfunction could reflect the altered molecular interaction of MuRF1 with titin (Higashikuse et al., 2019). There is a MuRF1-binding site in titin adjacent to the titin kinase domain. Mutations of this region lead to hypertrophy and diastolic dysfunction in the medaka fish experimental model and Japanese patients with HCM associated with *TRIM63* (Higashikuse et al., 2019). In addition, mutations of the titin MuRF1-binding site lead to the expression shift to the stiffer titin isoforms, increased titin binding to MuRF1, and enhanced titin degradation through ubiquitination. Thus, MuRF1–titin interaction contributed to sarcomeric protein turnover and titin isoforms switch, determining muscle compliance and diastolic function (Higashikuse et al., 2019).

The development of systolic dysfunction has been reported as a distinct feature of *TRIM63*-associated cardiomyopathies (Salazar-Mendiguchía et al., 2020). Thus, Salazar-Mendiguchiá et al. demonstrated that *TRIM63*-homozygous HCM patients have significant degrees of the LGE phenomenon and progressed to LV systolic dysfunction more often than in the typical HCM, thereby representing a subgroup of increased risk of adverse events (Salazar-Mendiguchía et al., 2020). However, despite the marked degree of fibrosis reflected by LGE on MRI in both cases, no systolic dysfunction was noted in our patients. Possibly, this could be explained by the relatively young patient age, and a thorough follow-up within the next years will shed light on the frequency of systolic dysfunction in patients with *TRIM63*-associated cardiomyopathies and marked fibrosis.

MuRF1 tissue distribution raises the question of whether *TRIM63* mutations can cause skeletal myopathy. Olivé et al. reported a male patient with cardiac and skeletal myosin aggregate myopathy carrying the combination of homozygous *TRIM63* null-mutation and heterozygous *TRIM54* (encoding MuRF3) mutation (Olivé et al., 2015). Clinically, it presented as proximal muscle weakness and HCM with atrial flutter. Electron microscopy of muscle biopsy revealed, apart from I-bands and Z-discs disorganization, myosin-associated proteins aggregates and abnormal microtubules distribution in skeletal muscle cells. The latter demonstrated the possibility that MuRF1 and MuRF3 regulate not only sarcomere protein degradation but also spatial organization of the microtubules. However, whether the presence of both *TRIM63* and *TRIM54* variants or the homozygous *TRIM63* variant itself contributed to myopathic phenotype remains unclear (Olivé et al., 2015). Later, Jokela et al. described female patient with mild clinical symptoms of skeletal myopathy along with creatine kinase elevation and severe cardiac hypertrophy (in the absence of other diseases capable of producing the observed degree of hypertrophy) in association with *TRIM63*-homozygous variant alone (Jokela et al., 2019). Given that, the remarkable muscle hypertrophy of upper limbs observed in both patients potentially could reflect pseudohypertrophy due to the myopathic process. Of note, both patients performed sport and had, similar to the case presented by Olive et al., marked muscular hypertrophic phenotype (Olivé et al., 2015). Of note, Patient 1, who did sport with the prevalence of static load, displayed more severe hypertrophy. However, this notion needs further attention and deeper functional studies, including neuromyography and muscle MRI.

Our study has several important limitations. One of them is an inability to perform a genetic test on the parental DNA and identify whether three of four described variants are *de novo* or inherited. Another important limitation is an inability to verify in both cases if two detected variants belong to the same or different alleles. In light of the recently published data on the polygenic impact of many genetic variants into HCM, the role of these and other concomitant variants in the genes not yet described in connection to the observed phenotype cannot be excluded and may need further elucidation.

In summary, we have described two new patients with HCM due to the compound heterozygous *TRIM63* variants. Both patients presented with marked progressed myocardial hypertrophy and diastolic dysfunction from II to III grades and demonstrated clear indications for ICD implantation according to the accepted risk prediction models. Further data collecting regarding rare cases of compound *TRIM63* variants associated with inherited cardiac pathology will allow developing a more personalized approach in this rare cardiac disorder.

## Data Availability

The datasets presented in this study can be found in online repositories. The names of the repository/repositories and accession number(s) can be found in the article/Supplementary Material.
